# Coniferyl Ferulate, a Strong Inhibitor of Glutathione S-Transferase Isolated from Radix *Angelicae sinensis*, Reverses Multidrug Resistance and Downregulates P-Glycoprotein

**DOI:** 10.1155/2013/639083

**Published:** 2013-08-24

**Authors:** Chang Chen, Chuanhong Wu, Xinhua Lu, Zhiyong Yan, Jian Gao, Hui Zhao, Shaojing Li

**Affiliations:** ^1^Institute of Chinese Materia Medica, China Academy of Chinese Medical Sciences, Beijing 100700, China; ^2^New Drug Research and Development Centre of North China Pharmaceutical Group Corporation, Hebei 050015, China; ^3^College of Life Science and Engineering, Southwest Jiaotong University, Chengdu 610031, China; ^4^College of Pharmaceutical Science, Hebei University, Baoding, Hebei 071002, China; ^5^China Academy of Chinese Medical Sciences, Beijing 100700, China

## Abstract

Glutathione S-transferase (GST) is the key enzyme in multidrug resistance (MDR) of tumour. Inhibition of the expression or activity of GST has emerged as a promising therapeutic strategy for the reversal of MDR. Coniferyl ferulate (CF), isolated from the root of *Angelica sinensis* (Oliv.) Diels (Radix *Angelicae sinensis*, RAS), showed strong inhibition of human placental GST. Its 50% inhibition concentration (IC_50_) was 0.3 **μ**M, which was greater than a known GSTP1-1 inhibitor, ethacrynic acid (EA), using the established high-throughput screening model. Kinetic analysis and computational docking were used to examine the mechanism of GST inhibition by CF. Computational docking found that CF could be fully docked into the gorge of GSTP1-1. The further exploration of the mechanisms showed that CF was a reversible noncompetitive inhibitor with respect to GSH and CDNB, and it has much less cytotoxicity. Apoptosis and the expression of P-gp mRNA were evaluated in the MDR positive B-MD-C1 (ADR+/+) cell line to investigate the MDR reversal effect of CF. Moreover, CF showed strong apoptogenic activity and could markedly decrease the overexpressed P-gp. The results demonstrated that CF could inhibit GST activity in a concentration-dependent manner and showed a potential MDR reversal effect for antitumour adjuvant therapy.

## 1. Introduction

Chemotherapeutics provide the most effective treatment modality for metastatic cancer. However, resistance to anticancer chemotherapy remains a serious obstacle in cancer treatment. Primary and acquired resistance of tumour cells to anticancer drugs are major causes of the limited efficacy of chemotherapy [1, 2]. Tumours may be intrinsically drug resistant or develop resistance during treatment; a phenomenon that is known as multidrug resistance (MDR). Acquired resistance is particularly a problem as tumours not only become resistant to the drugs originally used in treatment but also become cross-resistant to other drugs [3].

Glutathione S-transferases (GSTs, EC 2.5.1.18) are a superfamily of Phase II detoxification enzymes that catalyse the conjugation of glutathione (GSH) to a wide variety of endogenous and exogenous electrophilic compounds including chemotherapeutic agents [4]. GSTs are present in human tissues and have been subdivided into at least eight gene-independent classes named Alpha, Pi, Mu, Theta, Zeta, Omega, Sigma, and Kappa. Resistant cells often have increased detoxification of compounds mediated by high levels of GSH and GST [5]. Evidence suggests that the GST isozymes may have additional functions beyond their catalytic role [6]. These roles might include protecting the cells from death, detoxifying chemotherapeutic agents, and inducing drug resistance by inactivating chemotherapeutic compounds via GSH conjugation [7]. Among these isoenzymes, overexpression of GSTP1-1 was found to be correlated with the resistance of some chemotherapeutic agents in human tumour cells including colon, stomach, pancreas, uterine cervix, breast, lung cancers, melanoma, and lymphoma [8, 9]. The activity of GST appears to be an important factor contributing to the resistance of tumour.

However, MDR to cancer chemotherapy is complex and may involve multiple mechanisms. Notably, a combination of mechanisms, rather than a sole mechanism, has often been observed in the resistance to antineoplastic drugs. A close correlation between the high activity of GSTP1-1 and overexpression of P-glycoprotein (P-gp) has often been found simultaneously in many MDR cell lines [10]. P-gp is a classical ABC transporter (the gene product of* ABCB1*/*MDR1*) and acts as an ATP-dependent active efflux pump for chemotherapeutic agents. P-gp-mediated MDR appears to be a major feature in drug resistance  [11–13]. Moreover, GSTP1-1 displays an additional antiapoptotic activity based on a protein-protein interaction with the c-Jun N-terminal kinase (JNK), a key enzyme in the apoptotic cascade [14], by which GST inhibitors seem to indirectly inhibit the abnormal expression of P-gp. Therefore, the therapeutic use of GST inhibitors is viable suggestions as MDR reversing agents to improve the efficacy of chemotherapy. Ethacrynic acid (EA) [15], an active diuretic, was one of the first generation of GST inhibitors to be utilised as chemosensitiser [16]. Since then, a variety of GST inhibitors focused on the substrate-binding site of the GST isozyme or glutathione analogue have currently been examined and found to modulate drug resistance by sensitising tumour cells to anticancer drugs [4, 17, 18]. Unfortunately, most of them, including EA, have not fared well in clinical trials due to poor efficacy and side effects [19]. 

Development of MDR reversing agents with higher activity and lower toxicity is a promising strategy in the battle against MDR as this approach could result in the enhanced efficacy of anticancer compounds. Natural products might be important sources as potential chemosensitising agents with greater inhibition of the activity of GST. However, only a few inhibitors of GSTP1-1 from natural products, such as quinine [20], thonningianin A [21], quercetin  [22], curcumin [23], and plant phenols [24], have been demonstrated to antagonise MDR in preclinical trials. 

The natural resources of Chinese medicine materials are abundant and diverse. In addition, these medicines have relatively few side effects in long-term clinical use. Accordingly, they should be good candidates for a new generation of GST inhibitors to modulate MDR. In this study, a high-throughput screening (HTS) model was established to screen for inhibitors of GST from natural Chinese herbs. Using this approach, a compound isolated from the roots of *Angelica sinensis* (Oliv.) Diels (Radix *Angelicae sinensis*, RAS) was found to be a strong inhibitor of GST. 

This compound was also investigated in inhibitory kinetic and computational docking to evaluate its mechanism of GST inhibition and the structure-activity relationship. Apoptosis analysis and a reverse transcription-polymerase chain reaction (RT-PCR) assay for P-gp/MDR1 expression in an Adriamycin-resistant human endometrial cancer cell line were also utilised to evaluate the ability of the compound to reverse MDR. The primary aim of this study was to provide natural source for the discovery of new drug candidate with MDR reversing activity.

## 2. Materials and Methods

### 2.1. Chemicals and Reagents

GST (mainly GSTP1-1, from human placenta), glutathione (GSH), 1-chloro-2, 4-dinitrobenzene (CDNB), RPMI-1640, 3′-(4,5-dimethylthiazol-2-yl)-2,5-diphenyl tetrazolium bromide (MTT), ethacrynic acid (purity ≥ 98%), RNase A, propidium iodide, and Adriamycin (purity ≥ 98%) were obtained from Sigma (St Louis, MO, USA). Coniferyl ferulate standard (purity ≥ 98%) was purchased for structure identification from Chengdu Herbpurify CO., LTD, Chengdu, China (QC number A-001-120726). TRIzol reagent and a RT-PCR assay kit were from Life Technologies (Carlsbad, California, USA). All other chemicals were from Beijing Chemical Co., Beijing, China. All the chemicals used were of analytical grade.

### 2.2. Cell Lines and Cell Culture

Human lung carcinoma A549 cells and human endometrial carcinoma B-MD-C1 cells were maintained in Dulbecco's modified Eagle's medium (DMEM, Life Technologies, Carlsbad, California, USA), supplemented with 10% foetal bovine serum (Takara Bio, Shimogyo-ku, Kyoto, Japan) and antibiotics (100 U/mL penicillin and 100 *μ*g/mL streptomycin). The drug-resistant cell line B-MD-C1 (ADR+/+) was a gift from Professor Baoen Shan; the cell line's multidrug resistance was maintained by culturing the cells at in 5 *μ*g/mL Adriamycin.

### 2.3. Plant Materials

RAS was collected in 2008 from the Gansu province of China, as identified by Professor Lanping Guo of the Institute of Chinese Materia Medica, China Academy of Chinese Medical Sciences. A voucher specimen (20080705024) was deposited in the Herbarium of the Institute of Chinese Materia Medica, China Academy of Chinese Medical Sciences.

### 2.4. Extraction and Isolation

**Figure 1:**
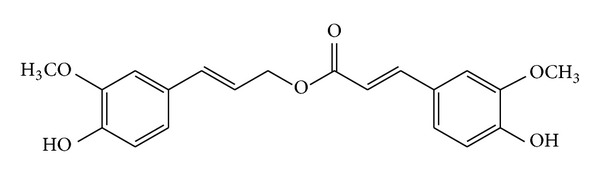
Structure of CF isolated from the Chinese herb RAS.

The isolation procedure for the studied compound was as follows. Briefly, dried RAS (10 kg) was ground into farina and extracted with 95% EtOH (100 L). After the insoluble farina was removed, the EtOH was evaporated under reduced pressure to give a viscous residue. The viscous residue was suspended in water and extracted with petroleum ether. The petroleum ether extract (400 g) was partitioned between petroleum ether and 80% MeOH, and the 80% MeOH layer (260 g) was chromatographed on a silica gel column (100 × 460 mm, 160–200 mesh). The column was eluted with a gradient of *n*-hexane/acetone from 90 : 10 to 10 : 90 to obtain seven fractions according to TLC detection. The fraction containing the compound was further separated by silica gel (column: 36 × 460 mm, 260~300 mesh, chloroform/acetone from 100 : 0 to 0 : 100), Sephadex LH20 column chromatography (column: 26 × 920 mm eluted with MeOH), and preparative HPLC (Phenomenex C18 column (21.2 × 250 mm, 10 *μ*m, 100 Å, Kromasil), eluted with 55% CH_3_CN-H_2_O) to give the test compound (249.0 mg). The compound was identical to one known compound, coniferyl ferulate (CF) [25]. Its purity was more than 95% by HPLC. The structure of the compound (Figure 1) was elucidated by analysis of mass spectrometry (MS) and ^1^H, ^13^C NMR data and confirmed with authentic sample (QC no. A-001-120726, Chengdu Herbpurify CO., LTD., Chengdu, China) spectra data of isolated compound CF as shown in supporting information in Supplementary Material available online at http://dx.doi.org/10.1155/2013/639083.

### 2.5. Enzyme Activity Assay

The inhibition studies using GST from human placenta were carried out at 37°C using the established HTS method in which GSH and CDNB are used as substrates [26]; GSH was used at 5 mM, and CDNB was used at 0.1 mM. One microlitre of the purified CF (final concentration from 0.1 *μ*M to 100 *μ*M) or positive control (final concentration from 0.1 *μ*M to 1 mM) dissolved in DMSO was added to the reaction mixture (10 *μ*L of GST, approximately 0.0018 units) dissolved in 50 mM potassium phosphate buffer (pH 7.4) in a 384-well plate. After incubation of a certain amount of GST and CF at 37°C for 30 min with GSH and CDNB, the change in absorbance at 340 nm was monitored to measure the product GSH conjugate formation with a spectrophotometer (Spectra Max M5, Molecular Devices, USA).

### 2.6. Enzyme Inhibitory Kinetics

Enzyme kinetic experiments were performed to elucidate the interaction of human GSTs with CF in detail. Firstly, the initial rate of the enzyme was analysed by measuring the formation of catalysate at 340 nm. The reaction was carried out at 37°C for 5 min after preincubating GST with various concentrations of CF for 5 min. Furthermore, a plot of *ν* (*μ*M/mL/min) versus [E] was obtained with different CF or EA concentrations (0%, 20%, and 50% inhibition rate to GST) and different GST concentrations of 0.0075–0.18 U/mL to distinguish between reversible and irreversible inhibition. A Hanes plot of *v* versus [S] was performed at GSH concentrations from 0.07 to 2.24 mM or CDNB concentrations from 0.5 to 44.1 *μ*M, with CF at concentrations of 0, 0.25, and 0.5 *μ*M.

### 2.7. Computational Docking Methods

Computational docking was performed with FlexX software in SBVS (rational drug design v7.0, Tripos Inc.). The protein data bank (PDB) file 2GSS and the cocrystal format of the inhibitor, EA, in the active site of GST (human GSTP1-1) were optimised and used. EA was selected as a reference ligand structure which was a fixed conformation docked into the active site of the enzyme. The flexible docking conformations of EA were then created with 30 docking conformation options. Each conformation was energy-minimised using a molecular mechanics program. All ligands were predicted based on the active sites being within a 6.5 Å radius from the bound ligand. Water and metals not involved in binding were removed from the protein. The docking scores in GSTP1-1 were employed and elucidated in detail.

### 2.8. Cytotoxic Activity by MTT Assay

The wild-type human endometrial cancer B-MD-C1 cells or the multidrug-resistant B-MD-C1 (ADR+/+) cells were plated in quadruplicate (6 × 10^3^ cells per well) and incubated in the presence of different concentrations of positive control EA (10 *μ*M–200 *μ*M) and CF (4.12 *μ*M–3 mM) for 48 h. The cytotoxic activity and dose response of EA and CF to these cell lines were determined using the MTT assay. Normalised data were plotted graphically as percentages of viable cells. Each study was performed in triplicate and repeated three times.

### 2.9. Flow Cytometry Apoptosis Analysis

Flow cytometry was used to evaluate the effect of CF on apoptosis of the multidrug-resistant B-MD-C1 (ADR+/+) cells treated with Adriamycin. Briefly, B-MD-C1 (ADR+/+) cells (1 × 10^7^/mL) were incubated with CF at various concentrations or positive control EA (40 *μ*m) for 48 h, and then the medium was replaced with fresh medium in the presence of Adriamycin (5 *μ*g/mL). The B-MD-C1 (ADR+/+) cells only treated with Adriamycin were used as a control. After incubation for 24 h at 37°C, the cells were collected and fixed in 70% cold ethanol (−20°C) overnight. The cell suspensions were washed twice with phosphate-buffered saline (PBS) and resuspended in phosphate-buffered saline containing 1% foetal calf serum. RNA in the fixed cells was digested with RNase A (0.5 mg/mL) at 37°C for 1 h. Finally, the cells were centrifuged at 1000 revolutions/min for 5 min. The cells were resuspended with binding buffer and stained with propidium iodide (2.5 *μ*g/mL) at room temperature for 15 min. The samples were measured by flow cytometry with CellQuest software (Becton Dickinson, San Jose, CA, USA).

### 2.10. MDR1 Gene Expression by RT-PCR

MDR1 gene expression levels were evaluated by RT-PCR assay on the B-MD-C1 and B-MD-C1 (ADR+/+) cells described above. Total cellular RNA was isolated using the TRIzol reagent (Life Technologies). First-strand cDNA synthesis was performed using a kit (Life Technologies). The primers used for the analysis of MDR1 were sense 5′-TCGTAGGAGTATCCGTGGAT-3′ and antisense 5′-CATTGGCGAGCCTGGTAG-3′ (455 bp); *β*-actin was used as an internal standard (sense primer 5′-AGCCCTTTCTCAAGGACCAC-3′ and antisense primer 5′-GCACTTTCTCCGCAGTTTCC-3′; 312 bp). The amplification reaction was carried out with 2 *μ*L of cDNA product for 35 cycles with each cycle consisting of 95°C for 30 sec, 55°C for 30 sec, and 72°C for 30 sec, followed by a final 5 min elongation at 72°C. The final RT-PCR products were visualised by ultraviolet illumination after electrophoresis through 1.5% agarose gel, with 0.5 mg/mL ethidium bromide at 50 V at 2 h, and scanned using Kodak gel analysis software. RNA amounts were normalised against the *β*-actin mRNA levels. EA (40 *μ*M) was also used as the positive control in this assay.

### 2.11. Statistical Analysis

Data were expressed as the means ± SD and analysed statistically using Student's *t*-test. The results were considered to be statistically significant when *P* < 0.05.

## 3. Results

### 3.1. The Inhibitory Activity of CF on GST

**Figure 2:**
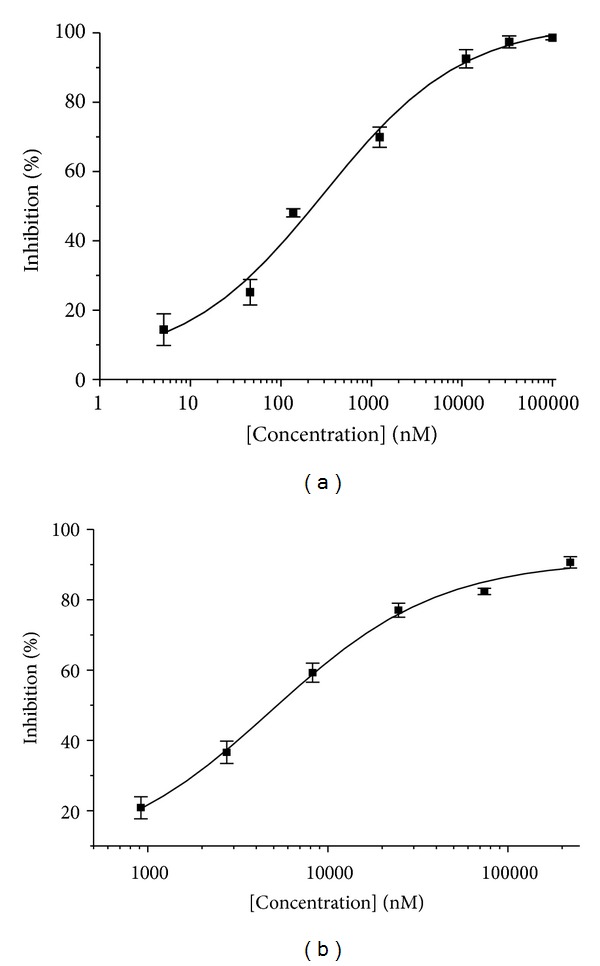
After incubation of GST and CF at 37°C for 30 min with 5 mM GSH and 0.1 mM CDNB, the rate of product formation was monitored by measuring the change in absorbance at 340 nm. The 50% inhibitory concentrations (IC_50_) were (a) CF, 0.30 *μ*M and (b) EA, 4.89 *μ*M, as a positive control.

**Figure 3:**
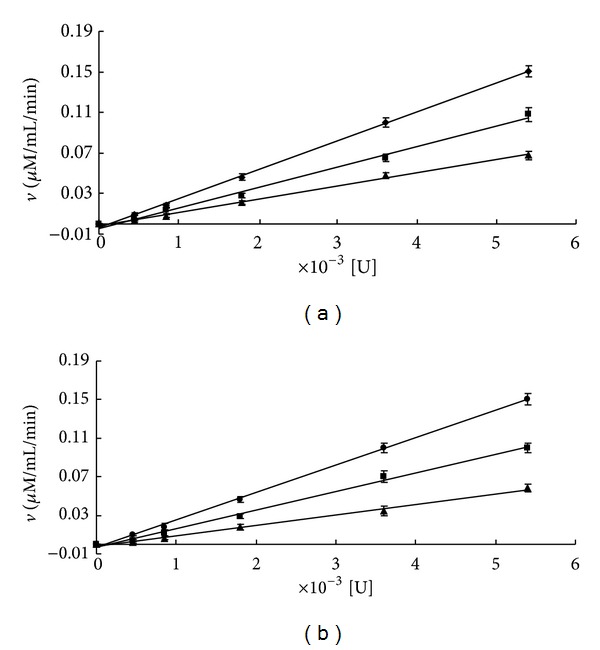
Kinetics plot of CF and EA to GST. The reaction was carried out at 37°C for 5 min after preincubating GST with CF for 5 min. A plot of *ν* (*μ*M/mL/min) versus [E] was obtained for GST concentrations from 0.0075 to 0.18 U/mL. Kinetics plots of inhibition to GST at 0%, 20%, and 50% IC for (a) CF (0 *μ*M, 0.25 *μ*M, and 0.5 *μ*M) and (b) EA (0 *μ*M, 2 *μ*M, and 5 *μ*M). Each point shows the mean ± SD of triplicate experiments.

**Figure 4:**
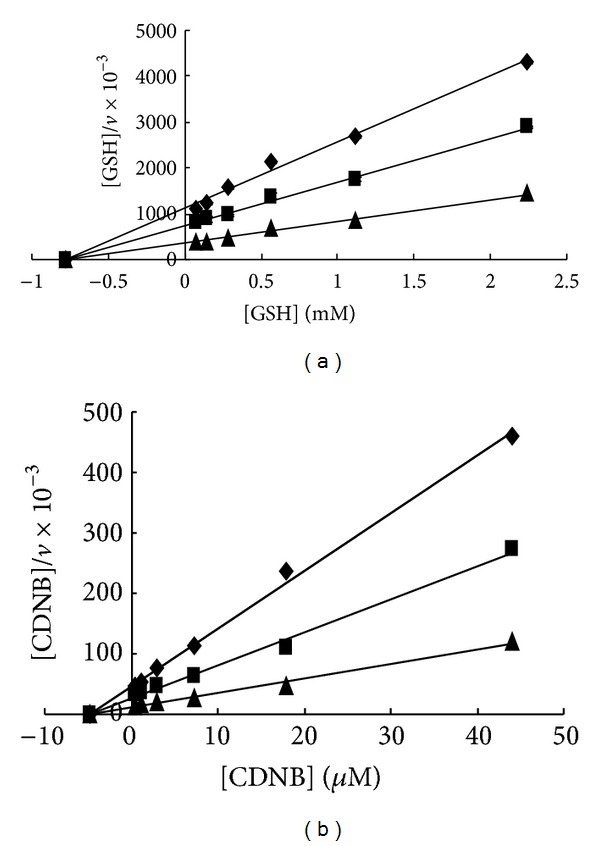
(a) A Hanes plot of *v* versus [S] for GSH. The reaction was carried out at 37°C for 5 min after preincubating GST with CF for 5 min. A Hanes plot of *v* versus [S] was created for GSH concentrations from 0.07 to 2.24 mM. (b) A Hanes plot of *v* versus [S] was created for CDNB concentrations from 0.5 to 44.1 *μ*M. The CF concentrations were 0 *μ*M, 0.25 *μ*M, and 0.5 *μ*M.

As shown in Figure 2, the inhibitory activity of CF on GST was investigated in the established HTS assay model. EA was chosen as a positive control that inhibited GST, with an IC_50_ value of 4.89 *μ*M (see Figure 2). Notably, CF showed the stronger inhibition of GST (mainly GST-pi, from human placenta) in a concentration-dependent manner. The 50% inhibitory concentration (IC_50_) of CF was approximately 0.3 *μ*M. Moreover, the inhibitory kinetics and the associated binding mode were investigated because they may provide an exploitable mechanism for developing potent drugs with desirable properties. The kinetic analysis of the mechanism of inhibition to GST by CF was investigated by varying either GSH or CDNB concentration. The results showed that the inhibition of GST by CF might be reversible (Figure 3).  Figure 4 shows the Hanes plots with various concentrations of GSH or CDNB. The results of the Hanes plots indicated that CF inhibited GST noncompetitively (Michaelis constant [*K*_*m*_] remained unchanged, whereas the maximum rate of clearance [*V*_max⁡_] decreased) with respect to GSH and CDNB. Therefore, CF was likely to act as a reversible noncompetitive inhibitor of GST.

### 3.2. Computational Docking for CF

**Figure 5:**
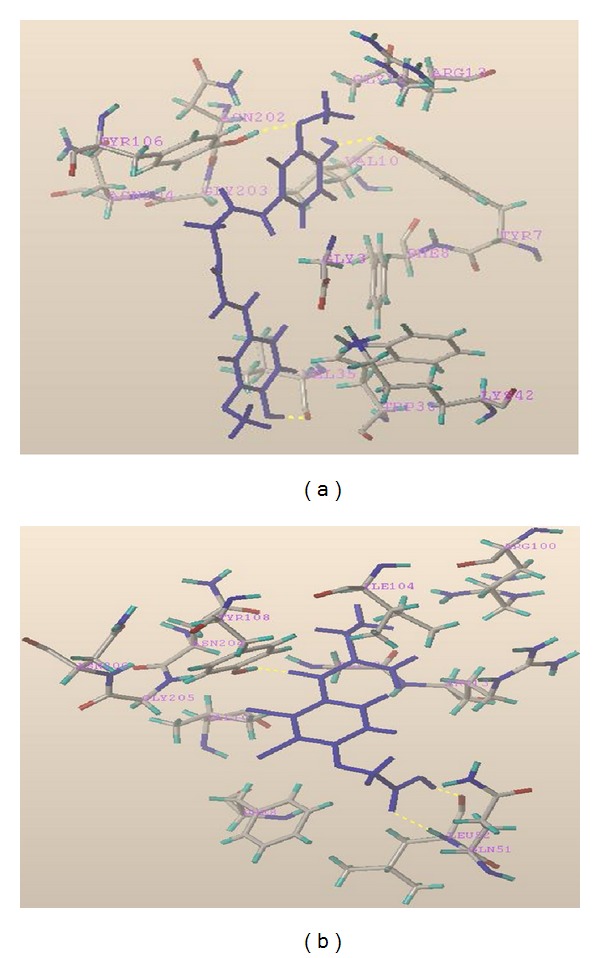
Computational docking with PDB file 2GSS as a reference structure. (a) CF hydroxyl oxygen atoms in a phenyl ring formed a hydrogen bond with active site amino acid TYR7. Methoxy oxygen atoms in the same phenyl ring formed a hydrogen bond with active site amino acid TYR106. Hydroxyl hydrogen atoms in another phenyl ring formed a hydrogen bond with the carbonyl oxygen atom of VAL35 in the active site. The conformation score was −11.0. (b) Ethacrynic acid's carbonyl oxygen atom forms a hydrogen bond with active site amino acid TYR108. The carboxyl oxygen atom forms a hydrogen bond with active site amino acid LEU52, and the hydroxyl oxygen atom forms a hydrogen bond with active site amino acid GLN51. The conformation score is −13.3.

In order to gain an insight into the structural basis by which CF exerts its inhibitory activity on GST, the active site of GST was analysed using the GST-EA complex, and molecular docking studies were performed on CF using the PDB file 2GSS as a reference structure. The computational docking method was used with FlexX software to dock CF into the active site of GST. This method demonstrated that CF might insert into the enzyme molecule active cavity but not bind to the active site. A compound with a high-scoring conformation would display a high inhibitory activity on GST, and the computational docking scoring conformations were consistent with the results of the high-throughput screening. CF's hydroxyl oxygen atoms in the phenyl ring formed a hydrogen bond with active site amino acid TYR7. Methoxy oxygen atoms in the same phenyl ring also formed a hydrogen bond with active site amino acid TYR106. In addition, hydroxyl, hydrogen atoms in another phenyl ring formed a hydrogen bond with the carbonyl oxygen atom of active site amino acid VAL35. The conformation score was −11.0. The reference compound, EA, has a side chain carbonyl oxygen atom that forms a hydrogen bond with active site amino acid TYR108. The carboxyl oxygen atom forms a hydrogen bond with active site amino acid LEU52. The conformation score for the ethacrynic acid interaction is −13.3 (Figure 5).

### 3.3. The Cytotoxic Activities of CF

The cytotoxic activity of CF and positive control EA were analysed using the MTT assay. It was demonstrated that CF (3 mM or less) had no obvious influence on the proliferation of B-MD-C1 and B-MD-C1 (ADR+/+) cells (the inhibition rate of 3 mM was below 30%), while EA showed more potent inhibition of cell growth and induction of cell death than CF in these two cells (the inhibition rate of 60 *μ*M was above 45%). Our data showed that CF hardly had any cytotoxic effect on the wild-type B-MD-C1 and the B-MD-C1 (ADR+/+) cells when its dose was less than 3 mM (Table 1). However, from the results, greater than 40 *μ*M of EA seemed to affect cell viability, whereas 100 *μ*M EA significantly decreased viable cells and showed the inhibition rate of close to 100%. Considering the low cytotoxicity, 5, 10, and 20 *μ*M CF and 40 *μ*M EA were chosen as reversal agents in our further study.

### 3.4. The Reversal Effect of CF on Cancer MDR Cell Lines

#### 3.4.1. The Effect of CF on Apoptosis

**Figure 6:**
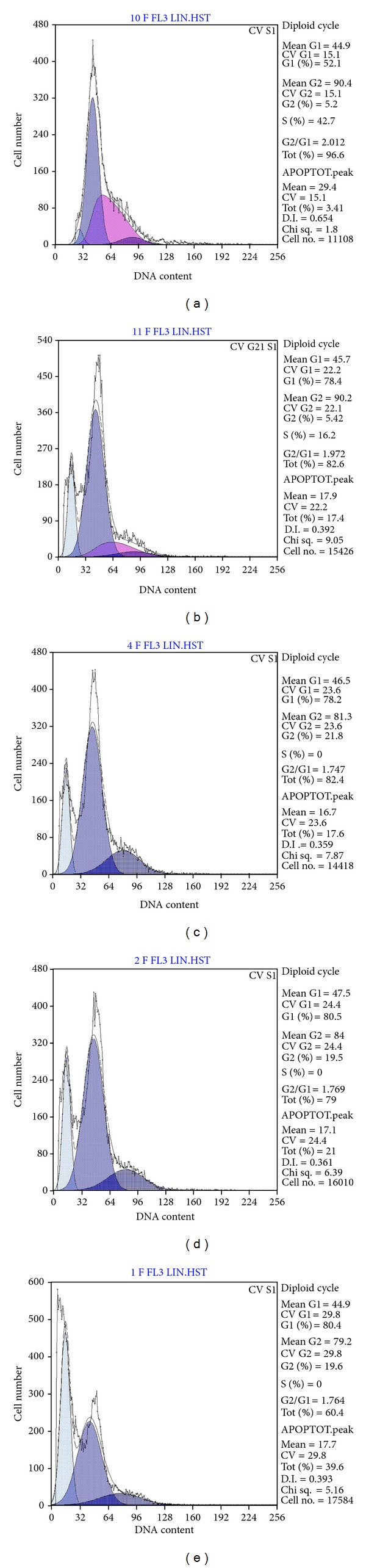
Flow cytometry results for the effect of CF on apoptosis of B-MD-C1 (ADR+/+) cells induced by Adriamycin. (a) Control; (b) EA concentration of 40 *μ*M; CF concentrations of (c) 5 *μ*M, (d) 10 *μ*M, and (e) 20 *μ*M.

To further investigate the reversal effect of CF on cancer MDR cell lines, its effects were determined on apoptosis of B-MD-C1 (ADR+/+) cells by flow cytometry. After the cells were exposed to 5 *μ*g/mL Adriamycin together with various concentrations of CF or 40 *μ*M EA for 48 h, a distinct sub-G1 peak, the apoptotic fraction, was observed in the cells compared with the control (Table 2, Figure 6). B-MD-C1 (ADR+/+) cells showed gradual arrest in the G(1)/S phase with the increasing doses of CF. Apoptosis induced by CF markedly increased the proportion of cells in G1-phase, decreased the proportion of cells in S-phase, and decreased cell survival. The apoptosis-inducing capacity of CF was much stronger than the positive control EA in this assay.

#### 3.4.2. The Effect of CF on P-gp Expression Level

**Figure 7:**
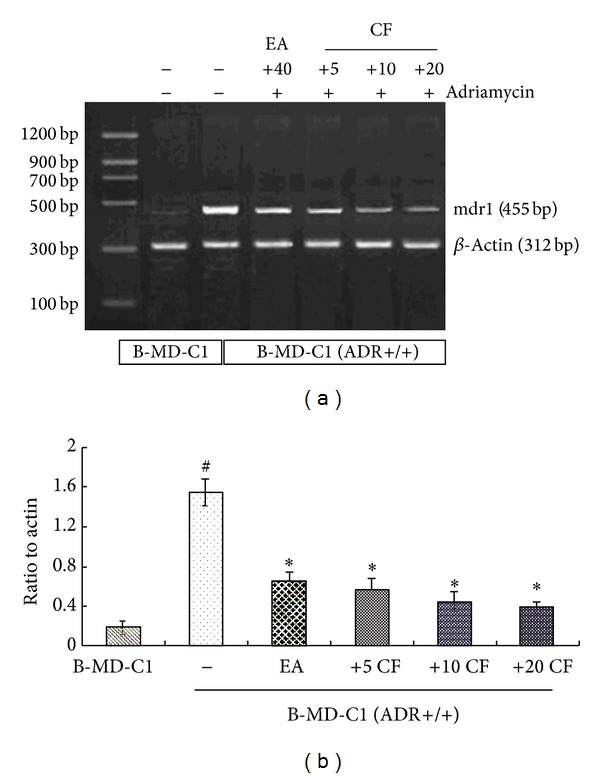
Effect of CF on MDR1 expression in B-MD-C1 (ADR+/+) cells. Cells were incubated with various concentrations of CF (5 *μ*M, 10 *μ*M, and 20 *μ*M) or Adriamycin (5 *μ*g/mL) for 48 h. EA (40 *μ*M) as positive control. MDR1 bands were noted in (a). Densitometric analysis is shown in (b). ^#^ *P* < 0.01 B-MD-C1 versus B-MD-C1 (ADR+/+), *P < 0.01 compound (+)/Adriamycin (+) versus compound (−)/Adriamycin (+) group. All the data shown are representative of three independent experiments.

RT-PCR analysis of the expression level of P-gp in the studied groups further revealed the effect of CF on reversing MDR in MDR-positive cells (Figure 7(a), lane 1). MDR1/P-gp was significantly increased with exposure to Adriamycin (Figure 7(a), lane 2). This increased level of MDR1/P-gp with exposure to Adriamycin was found to be reversed to normal levels by treatment with various concentrations of CF (Figure 7(a), lanes 3, 4, and 5), and the P-gp levels were significantly decreased in groups pretreated with Adriamycin and CF at various concentrations compared with the group only treated with Adriamycin (*P* < 0.01, Figure 7(b)). CF seemed to be more potent in the inhibition of P-gp expression than the positive control EA.

## 4. Discussion

Conventional cancer chemotherapy is seriously limited by MDR commonly exhibited by tumour cells. MDR has been recognised as an important type of resistance that can be due to various mechanisms. These various mechanisms might explain why treatment regimens that combine multiple agents with different targets are less effective than expected [11, 27, 28]. 

Of these mechanisms, one of the most frequently encountered mechanisms for the acquisition of MDR by tumour cells is the induction and activation of efflux transporter proteins. P-gp is commonly accepted as one of the best characterized transporters responsible for the multidrug resistance phenotype exhibited by cancer cells. Most of the researchers pay more attention to find molecules that can directly block the activity of P-gp, which is a common step and a well-accepted strategy to reverse MDR phenotype [29]. However, the characteristic structures of the P-gp, such as multiple active binding sites, too large protein molecule (consists of 12 transmembrane domains, and two cytoplasmic ATP-binding domains), make it so difficult to evaluate activity in vitro and find more effective single-target candidate compound. And as an important transporter protein, it is believed that P-gp may play a significant role in the processes of drug absorption, distribution, metabolism, and excretion and may protect the healthy human body against toxic xenobiotics by excreting these compounds into the bile, urine, and the intestinal lumen and by preventing their accumulation in the brain. P-gp is also closely related with the drug-metabolizing enzymes, such as CYP 3A4 [30]. So developing drug candidate which is a possible strong P-gp modulator may most likely cause the potential side effects [28]. 

Cancer cells can also acquire resistance by overexpressing GSTs that may increase detoxification and circumvent the cytotoxic action of antitumour drugs. In particular, a number of chemotherapy agents currently used in cancer therapy are known to be substrates of GSTs [31], and it has been clearly shown that overexpression of GSTs in tumours is closely linked to the development and expression of MDR [4]. The GST superfamily, particularly the P1-class GST (GSTP1-1), is frequently overexpressed in various human cancers. GST is obviously a key resistance factor for anticancer drugs and has become the focus of extensive pharmaceutical research in an attempt to generate more efficient anticancer agents [32]. Therefore, GST is a valuable target for the development of inhibitors that could be used to increase chemotherapeutic efficiency and to address MDR. Moreover, accumulating studies suggest that MDR is closely correlated with the combination action of the high level of GSTs and overexpression of P-gp and the increased GST activity could influence the expression of P-gp via several signal pathways. GSTP1-1 activity and P-gp levels were often found higher in the chemotherapy-resistant cancer cell lines, such as the B-MD-C1 cell line treated with Adriamycin compared to the sensitive cells. Exposure to Adriamycin rapidly increased GST activity and P-gp expression in the resistant cells [33, 34]. So it could be a better way to find new MDR reversal agents by screening for inhibitors of GST, which can indirectly regulate P-gp, rather than having a direct effect on the P-gp.

Natural products should be a noteworthy resource for the generation of potential MDR reversal agents that have higher levels of inhibition of GST. However, the GST inhibitors from natural sources have not been well studied, only a few natural components have been examined in clinical trials, and their efficacy has been less than expected. There is our interest in finding the strong GST inhibitors from natural products and then observing the effect of the active compound on reversing MDR. RAS, known as Chinese Angelica, is the root of *Angelica sinensis *(Oliv.) Diels, which has been used in traditional Chinese medicine (TCM) for more than 2000 years [35]. It has been mostly used as one of the herbal ingredients in prescriptions of TCM to treat gynaecological diseases. However, more and more studies show that RAS has a variety of pharmacological activities, including antitumour activity [36, 37]. 

Two ligustilide compounds isolated from RAS have been reported to be the inhibitors of GST in a previous study from our laboratory [26]. In this study, another compound, CF, also isolated from RAS, showed a stronger GST inhibitory effect than the positive control EA. EA is one of the first generation GST inhibitors that was utilised as a classical MDR reversal agent, and it inhibits GST-Alpha, -Mu, and -Pi by binding directly to the substrate binding site of these isozymes. In addition, EA inhibits GST by depleting its cofactor, GSH, via conjugation of the Michael addition intermediate to the thiol group of GSH [15, 38, 39]. An enzyme inhibitory kinetic analysis was conducted in some detail to clarify the mechanism of inhibition of GST by CF. The results gave the hint that the inhibitory binding site of CF might not be the catalytic sites. CF did not compete with GSH for the GSH-binding site (G-site) nor did it compete with CDNB for the CDNB-binding site (H-site). These effects suggested that CF induced conformational changes and hence made enzyme inactivation. The kinetic characteristics showed a valuable property of CF.

This study also investigated the structure-activity relationship and the molecule's binding mode to understand the pharmacologic action of this compound. Docking is one of the most commonly used techniques in drug design. It is used for both identifying correct poses of a ligand in the binding site of a protein as well as for the estimation of the interactions between potential drugs and the target proteins. In this work, a docking analysis using FlexX, combined with the HTS of human GST inhibitors, was employed to identify the effect of the inhibitor on the structure-activity relationship. It was found that CF could be fully docked into the gorge of GSTP1-1 and that hydrogen bond interactions could be an important factor to the binding affinity of CF in the active cavity. In addition, these interactions might be important for the inhibition of GST through a conformation change. 

Moreover, in order to clarify whether CF itself could make the influence on the growth of the test tumour cells, the cytotoxicity assays were performed on the MDR phenotype B-MD-C1 (ADR+/+) and wild-type B-MD-C1 cell lines. The results showed much less cytotoxicity with CF treatment alone compared to EA alone. Based on the high GST inhibitory activity and the low cytotoxicity, the further apoptosis analysis by flow cytometry and RT-PCR analysis of MDR1 gene expression had been implemented to evaluate the MDR reversal effect of CF. The B-MD-C1 (ADR+/+) cells presented the typical acquired Adriamycin resistance with the features of high activity of GST and overexpression of P-gp. The flow cytometry data demonstrated a strong apoptogenic activity when the cells were treated with a certain concentration of Adriamycin and CF together. CF, in a concentration-dependent manner, significantly induced apoptosis in the B-MD-C1 (ADR+/+) cells and altered the phase distribution of cell cycle. RT-PCR analysis showed that the overexpression of P-gp when the cells exposed to Adriamycin was markedly decreased by CF. CF might inhibit the P-gp expression and thus increase the intracellular Adriamycin accumulation. It was of interest that CF seemed to show much stronger effect of Adriamycin resistance reversal than the positive control EA in our study. 

## 5. Conclusions

In conclusion, we demonstrated the strong GST inhibitory activity and the action mechanism of CF, the component from the Chinese medicine RAS. These studies add powerful novel evidence that RAS was a potential source to provide an effective MDR reversal agent for cancer. The compound CF could also be used as a promising lead compound for chemosensitization that was able to indirectly regulate P-gp expression via modulation of GST activity with the possible lower adverse effect and warrants further investigation in antitumour adjuvant therapy. 

## Supplementary Material

In order to identify the molecular structure of coniferyl ferulate (CF), the mass spectrum [TOF-MS spectra (ES+ and ES-) of CF] and nuclear magnetic resonance spectroscopy [1H-NMR spectrum (500 MHz) of CF in CDCl3 and 13C-NMR spectrum (125 MHz) of CF in CDCl3] had been provided as the supporting information.

## Figures and Tables

**Table 1:** The inhibitory effect of CF and positive control EA on the proliferation of B-MD-C1 and multidrug-resistant B-MD-C1 (ADR+/+) cells ($\bar{X}\pm \text{SD}$, *n* = 3, 48 h).

CF (*μ*M)	Inhibitory rate (%)		EA (*μ*M)	Inhibitory rate (%)	
	B-MD-C1	B-MD-C1 (ADR +/+)		B-MD-C1	B-MD-C1 (ADR+/+)
3000	27.39 ± 4.70	22.67 ± 7.49	200	99.29 ± 5.68	99.89 ± 7.76
1000	18.62 ± 2.87	10.72 ± 3.21	100	99.52 ± 7.49	94.68 ± 10.23
333.33	5.07 ± 0.20	6.33 ± 0.35	80	78.72 ± 4.53	85.56 ± 5.62
111.11	5.30 ± 1.45	5.42 ± 2.20	60	53.30 ± 6.40	45.61 ± 4.43
37.04	2.56 ± 0.51	3.86 ± 0.49	40	6.56 ± 0.89	5.09 ± 1.35
12.35	1.36 ± 0.41	2.56 ± 0.27	20	4.01 ± 1.18	2.14 ± 1.28
4.12	1.02 ± 0.35	2.11 ± 0.45	10	3.02 ± 0.86	1.34 ± 0.57

**Table 2:** Flow cytometry data for the effect of CF on apoptosis of B-MD-C1 (ADR+/+) cells ($\bar{X}\pm \text{SD}$, *n* = 3).

Treatment	G1%	S%	Apoptosis (%)
Control	57.30 ± 7.26	41.87 ± 7.39	4.82 ± 1.38
EA, 40 *μ*M	76.63 ± 4.23*	6.07 ± 3.86*	16.83 ± 4.69*
CF, 5 *μ*M	78.77 ± 3.48*	0*	17.13 ± 3.52*
10 *μ*M	80.43 ± 5.00*	0*	21.73 ± 3.36*
20 *μ*M	81.20 ± 5.15*	0*	39.40 ± 5.20*

Values are presented as the mean ± SD (*n* = 3). *P < 0.01 versus control.
