# Supplementary figures and images for: Coniferyl Ferulate, a Strong Inhibitor of Glutathione S-Transferase Isolated from Radix Angelicae sinensis, Reverses Multidrug Resistance and Downregulates P-Glycoprotein
Source: Evid Based Complement Alternat Med. 2013 Aug 24;2013:639083. doi: 10.1155/2013/639083 (PMC3766616; doi:10.1155/2013/639083)

TOF-MS spectra (ES+ and ES-) of CF


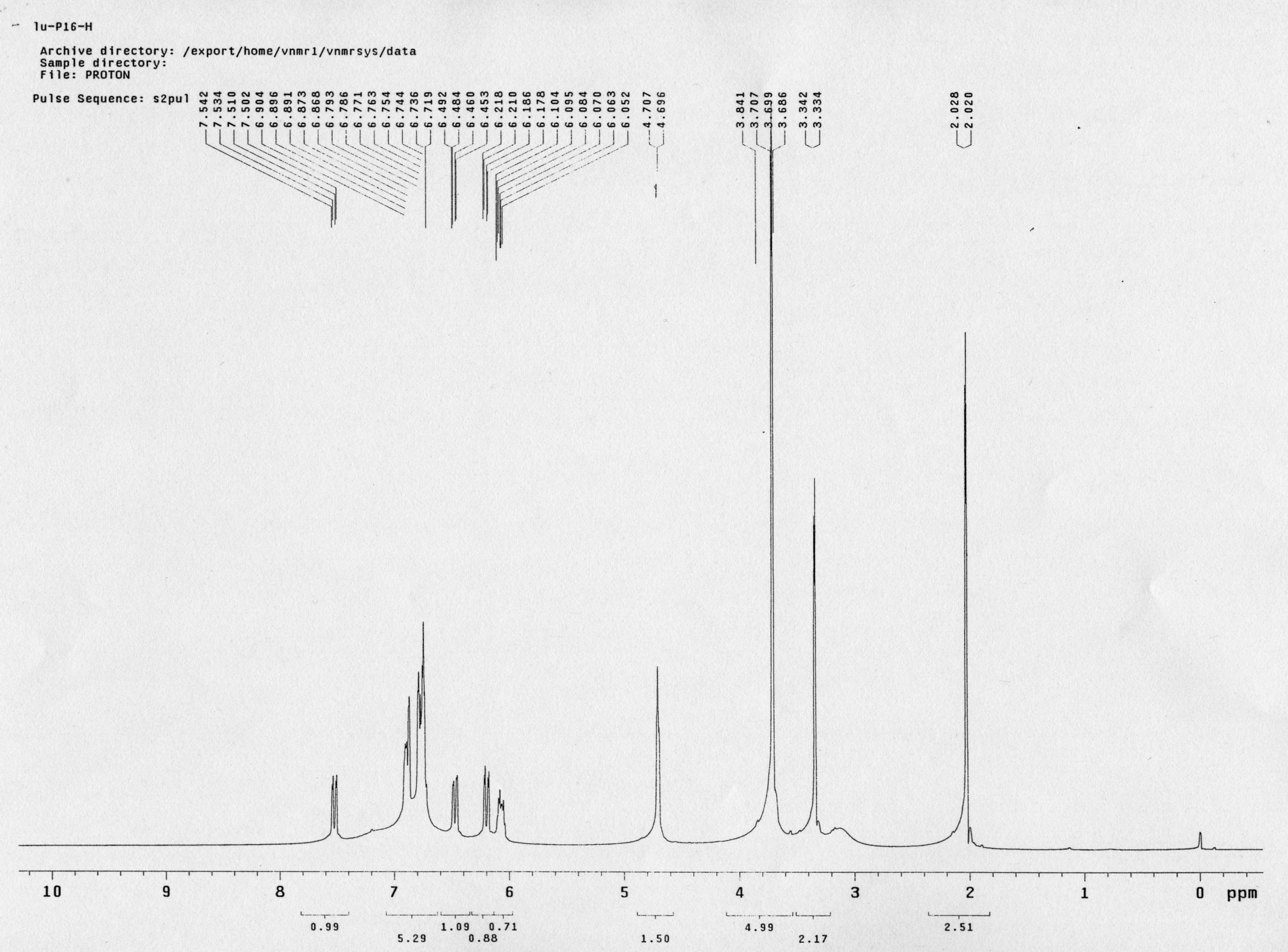


1H-NMR spectrum (500 MHz) of CF in CDCl3


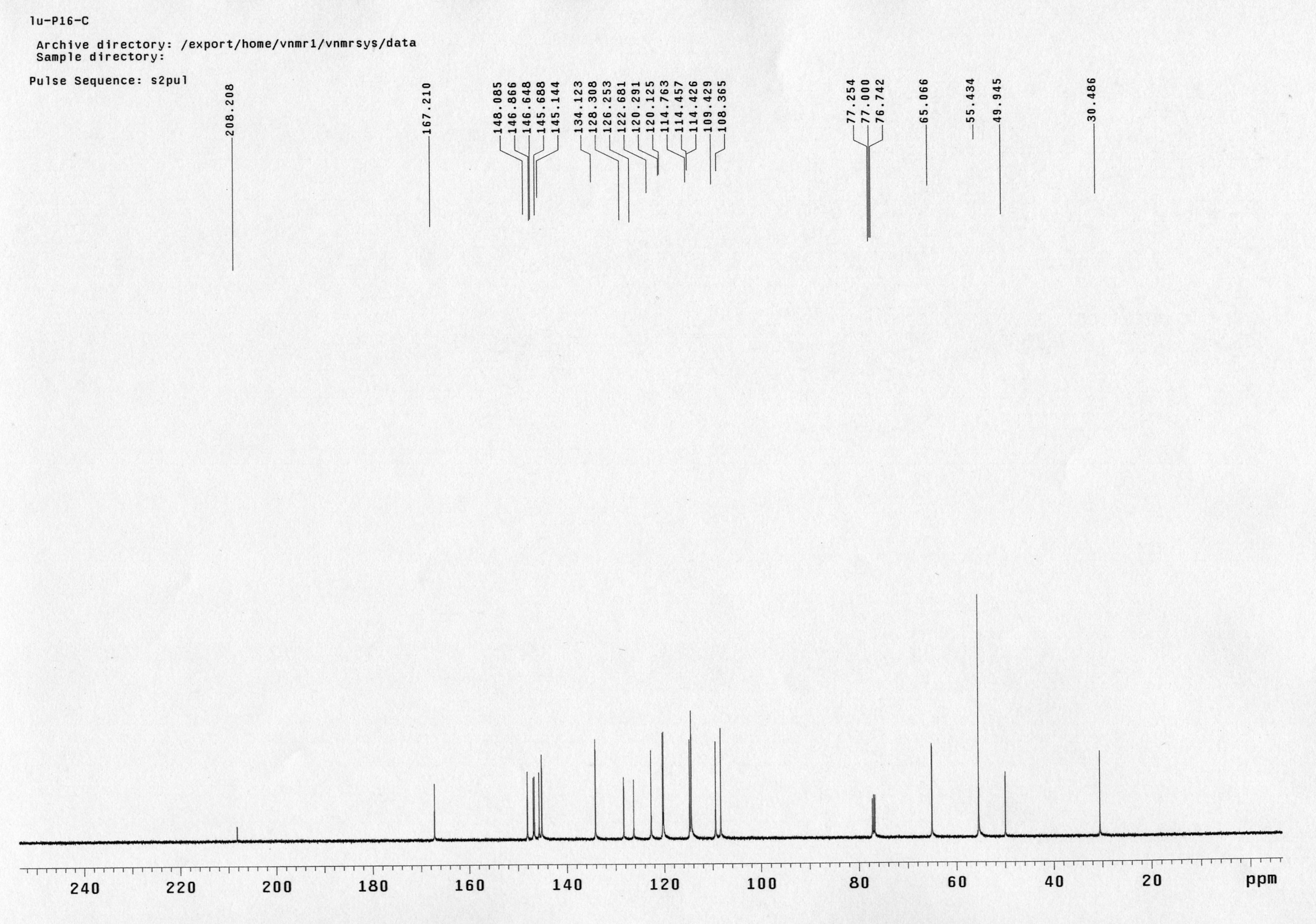


13C-NMR spectrum (125 MHz) of CF in CDCl3

Supplement: Supplementary file 1 — In order to identify the molecular structure of coniferyl ferulate (CF), the mass spectrum [TOF-MS spectra (ES+ and ES-) of CF] and nuclear magnetic resonance spectroscopy [1H-NMR spectrum (500 MHz) of CF in CDCl3 and 13C-NMR spectrum (125 MHz) of CF in CDCl3] had been provided as the supporting information. [file 639083.f1.doc]
